# Circulating MicroRNA Expression Profiles in Patients with Stable and Unstable Angina

**DOI:** 10.6061/clinics/2020/e1546

**Published:** 2020-07-06

**Authors:** Sudong Liu, Xuemin Guo, Wei Zhong, Ruiqiang Weng, Jing Liu, Xiaodong Gu, Zhixiong Zhong

**Affiliations:** I Center for Cardiovascular Diseases, Meizhou People's Hospital (Huangtang Hospital), Meizhou Hospital Affiliated to Sun Yat-sen University, Meizhou 514031, P. R. China; II Center for Precision Medicine, Meizhou People's Hospital (Huangtang Hospital), Meizhou Hospital Affiliated to Sun Yat-sen University, Meizhou 514031, P. R. China; III Research Experimental Center, Meizhou People's Hospital (Huangtang Hospital), Meizhou Hospital Affiliated to Sun Yat-sen University, Meizhou 514031, P. R. China; IV Guangdong Provincial Key Laboratory of Precision Medicine and Clinical Translational Research of Hakka Population, Meizhou 514031, P. R. China

**Keywords:** Coronary Artery Disease, MicroRNA (miRNA), Stable Angina, Unstable Angina, RNA sequencing (RNA-seq)

## Abstract

**OBJECTIVES:**

High incidence and case fatality of unstable angina (UA) is, to a large extent, a consequence of the lack of highly sensitive and specific non-invasive markers. Circulating microRNAs (miRNAs) have been widely recommended as potential biomarkers for numerous diseases. In the present study, we characterized distinctive miRNA expression profiles in patients with stable angina (SA), UA, and normal coronary arteries (NCA), and identified promising candidates for UA diagnosis.

**METHODS:**

Serum was collected from patients with SA, UA, and NCA who visited the Department of Cardiovascular Diseases of the Meizhou People’s Hospital. Small RNA sequencing was carried out on an Illumina HiSeq 2500 platform. miRNA expression in different groups of patients was profiled and then confirmed based on that in an independent set of patients. Functions of differentially expressed miRNAs were predicted using gene ontology classification and Kyoto Encyclopedia of Genes and Genomes pathway analysis.

**RESULTS:**

Our results indicated that circulating miRNA expression profiles differed between SA, UA, and NCA patients. A total of 36 and 161 miRNAs were dysregulated in SA and UA patients, respectively. miRNA expression was validated by reverse transcription quantitative polymerase chain reaction.

**CONCLUSION:**

The results suggest that circulating miRNAs are potential biomarkers of UA.

## INTRODUCTION

Coronary artery disease (CAD) is a major global health problem (1), and the acute coronary syndrome (ACS), which is the most severe form of CAD, is the leading cause of death and disability worldwide (2,3). Early and accurate diagnosis of ACS is critical for effective treatment, for reducing cardiovascular events, and for improving disease prognosis.

CAD is a chronic inflammation which comprises several stages from chest pain to stable angina (SA) which may aggravate to more severe unstable angina (UA) or acute myocardial infarction (AMI) (4). AMI is diagnosed by angiography and plasma biomarkers such as cardiac troponin (5). However, relatively few specific biomarkers are available to distinguish UA from SA, which is critical, as UA is more likely to produce poor outcomes. Therefore, novel clinical biomarkers for early diagnosis of UA are required.

MicroRNAs (miRNAs) are endogenous RNAs with a length of approximately 22-24 nucleotides which post-transcriptionally control gene expression by targeting the 3′-untranslated region of mRNA (6). It is estimated that miRNAs regulate about 60% of protein-coding genes that are involved in various biological processes such as cell growth, differentiation, and apoptosis (7). Many recent studies suggest that miRNAs play crucial roles in pathophysiologic processes of cardiovascular diseases (8-10). Moreover, miRNA expression is associated with atherosclerotic plaque stability (11). miRNAs are tissue-specific and are traceable in the circulatory system, which makes them suitable as diagnostic biomarkers (12-14). Several studies suggested that certain miRNAs, i.e., miR-1, miR133a, miR-133b, and miR-499, are dysregulated in patients with cardiac injury and myocardial infarction (15-17). A different study showed that circulating miRNAs are promising diagnostic and prognostic UA biomarkers (18). However, only few biomarkers can be used to reliably distinguish UA from SA; therefore, research on miRNA candidates is needed to identify useful biomarkers for UA diagnosis.

In the present study, we examined distinct miRNA profiles in plasma of SA and UA patients using high-throughput sequencing. The objective of this study was to identify specific miRNAs that can be used as biomarkers of UA.

## MATERIALS AND METHODS

### Study subjects

Subjects were recruited from all patients who attended the Department of Cardiovascular Diseases of the Meizhou People’s Hospital between January 1, 2017, and December 31, 2018. The study cohort included three groups, i.e., UA patients, SA patients, and patients with normal coronary arteries (NCA). Patients were diagnosed angiographically with CAD if at least one major epicardial vessel showed >50% stenosis. CAD patients were categorized as UA patients according to the 2014 AHA/ACC guidelines for management of ACS patients (19), or as SA patients according to the ACC/AHA guidelines (20). Patients with no observable stenosis in the coronary artery, as determined by coronary angiography, were considered NCA patients. Clinical history and medication records of patients were compiled, and subjects were excluded if they had a left ventricular ejection fraction of less than 45% or if they suffered from congestive heart failure, severe infectious diseases, or other malignant diseases.

### Ethics approval

The study was approved by the Ethical Committee of the Meizhou People’s Hospital. Written informed consent was obtained from each subject.

### Sample collection

Peripheral venous blood samples were collected after coronary angiography surgery. Blood samples were placed in EDTA-coated tubes. Plasma was separated by centrifugation at 1600 *g* and 4°C for 10 min. Supernatants were transferred to new tubes which were then centrifuged at 14,000 *g* and 4°C for 15 min to produce cell- and platelet-free plasma samples which were then stored at -80°C until further use.

### RNA extraction

RNA was isolated from plasma using a QIAamp Circulating Nucleic Acid kit (Qiagen, Valencia, CA, USA) following the manufacturer’s instructions. The concentration and purity of the miRNAs were assessed using a NanoDrop-1000 spectrophotometer (Thermo Fisher Scientific, Waltham, MA, USA). For normalization, *Caenorhabditis elegans* miR-39 (cel-miR-39) was added to plasma samples after addition of TRIzol, according to previously published study (21).

### Small RNA sequencing

A total of 3 μg of qualified RNA was used for miRNA library preparation using a TruSeq Small RNA Library Preparation Kit (Illumina, San Diego, CA, USA). Briefly, 3′- and 5′-adaptors were ligated to miRNAs, followed by reverse transcription (RT) using RT primers. Complementary DNA (cDNA) was amplified, and the products were separated using polyacrylamide gel electrophoresis. Fragments containing 140-160 bp were recovered to produce cDNA libraries. Library concentration was measured using a Qubit 2.0 fluorometer (Thermo Fisher Scientific). cDNA libraries were sequenced on a HiSeq 2500 platform (Illumina). Raw reads were decoded and annotated as small RNAs and sequence data were bioinformatically analyzed according to established methods (22).

### RNA sequence data analysis

Raw reads in fastq format were processed to remove low-quality reads, ambiguous nucleotides, adapter sequences, and poly-A tails to retain clean reads only. Pre-processed miRNA datasets were mapped to miRNA reference sequences using miRBase R19 software. Expression values of specific miRNAs were calculated as transcripts per million reads (TPM), according to the following equation:



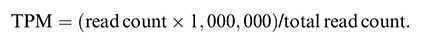



### Target gene analysis

Putative targets of dysregulated miRNAs were predicted using the three databases miRanda, MicroCosm, and Targetscan. To improve prediction accuracy, we chose putative targets that were identified in all three databases. To further explore potential biological functions of miRNA targets, gene ontology (GO) terms classifications and Kyoto Encyclopedia of Genes and Genomes (KEGG) pathway analyses were performed. GO classification (regarding biological processes, cellular components, and molecular functions) was carried out using the software Database for Annotation Visualization and Integrated Discovery (42). Pathway analyses were performed using the KEGG database. In brief, enriched biological processes, cellular components, molecular functions, and signaling pathways were selected through hyper-geometric tests and Fisher’s tests after mapping the potential target genes to the dataset of GO terms and KEGG pathways. A false discovery rate <0.05 was considered statistically significant.

### Validation of miRNA expression

miRNA expression was detected using a Bulge-Loop miRNA qRT-PCR Starter Kit (RiboBio Co., Ltd, Guangzhou, China), according to the manufacturer’s instructions. Briefly, 1 μg miRNA was used for RT in a polymerase chain reaction (PCR) thermocycler at 42°C for 42 min and 70°C for 10 min. A quantitative real-time PCR (qPCR) reaction mix (20 ul) containing 2 ul RT product, 10 ul SYBR Green mix, 0.8 ul primers and 6.4 ul distilled water (RiboBio Co., Ltd, Guangzhou, China) was prepared, and amplification was carried out on a Roche LightCycler 480 instrument (Roche, Mannheim, Germany). cel-miR-39 was used as a spike-in reference, as is common practice (23,24). The delta cycle threshold (ΔCt) of miRNAs of interest was calculated using the following equation: ΔCt = Ct[miRNA of interest] - Ct[cel-miR-39]. miRNA expression was calculated using the 2^-ΔΔCt^ method with an arbitrary value of 1 for the control group.

### Statistical analyses

Data were analyzed using GraphPad Prism 5 software (GraphPad Software Inc., La Jolla, CA, USA). Student’s t-test was applied to test differences between two groups. A one-way ANOVA followed by Tukey’s post-hoc test was used to test differences between more than two groups; *p*-values <0.05 were considered statistically significant.

## RESULTS

### Clinical characteristics

Nineteen patient samples were subjected to small RNA sequencing. Basic clinical characteristics of these patients are shown in Table 1. No significant differences in these characteristics were observed between the study groups.

### Dysregulated miRNA expression profiles in UA and SA patients

**Figure 1:**
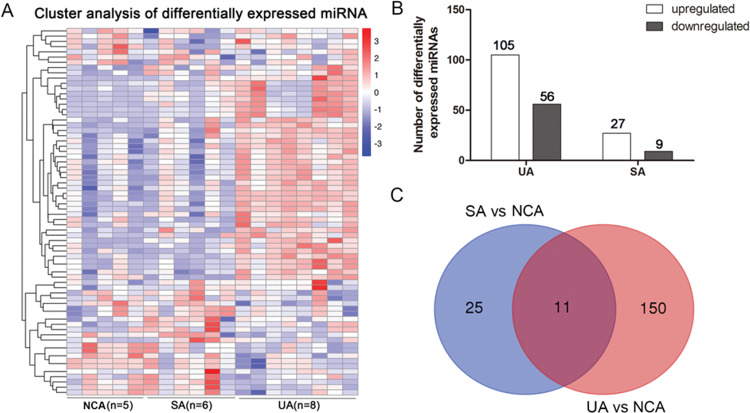
Differentially expressed miRNAs in patients with SA, UA, and NCA. (A) Cluster analysis of differentially expressed miRNAs in patients with SA, UA, and NCA. (B) Upregulated and downregulated miRNAs in patients with SA and UA. (C) Venn diagram of differentially expressed miRNAs in SA and UA patients compared with those of NCA patients. SA, stable angina (SA); UA, unstable angina; NCA, normal coronary artery.

A total of 1,314 miRNAs were identified, including 987 annotated and 327 novel miRNAs. A hierarchical cluster analysis indicated distinct miRNA expression profiles in UA, SA, and NCA patients (Figure 1A). To identify differentially expressed miRNAs, we used selection criteria of a fold change >2 and a *p*-value <0.05. A total of 161 miRNAs were dysregulated in UA compared to NCA, 105 of which were upregulated and 56 were downregulated; 36 miRNAs were differentially expressed in SA compared to NCA, 27 of which were upregulated and 9 were downregulated (Figure 1B). In total, 150 miRNAs were unique to the UA group, and 25 miRNAs were unique to the SA group. UA and SA groups shared 11 differentially expressed miRNAs (Figure 1C).

### GO functional analysis and KEGG pathway analysis

**Figure 2:**
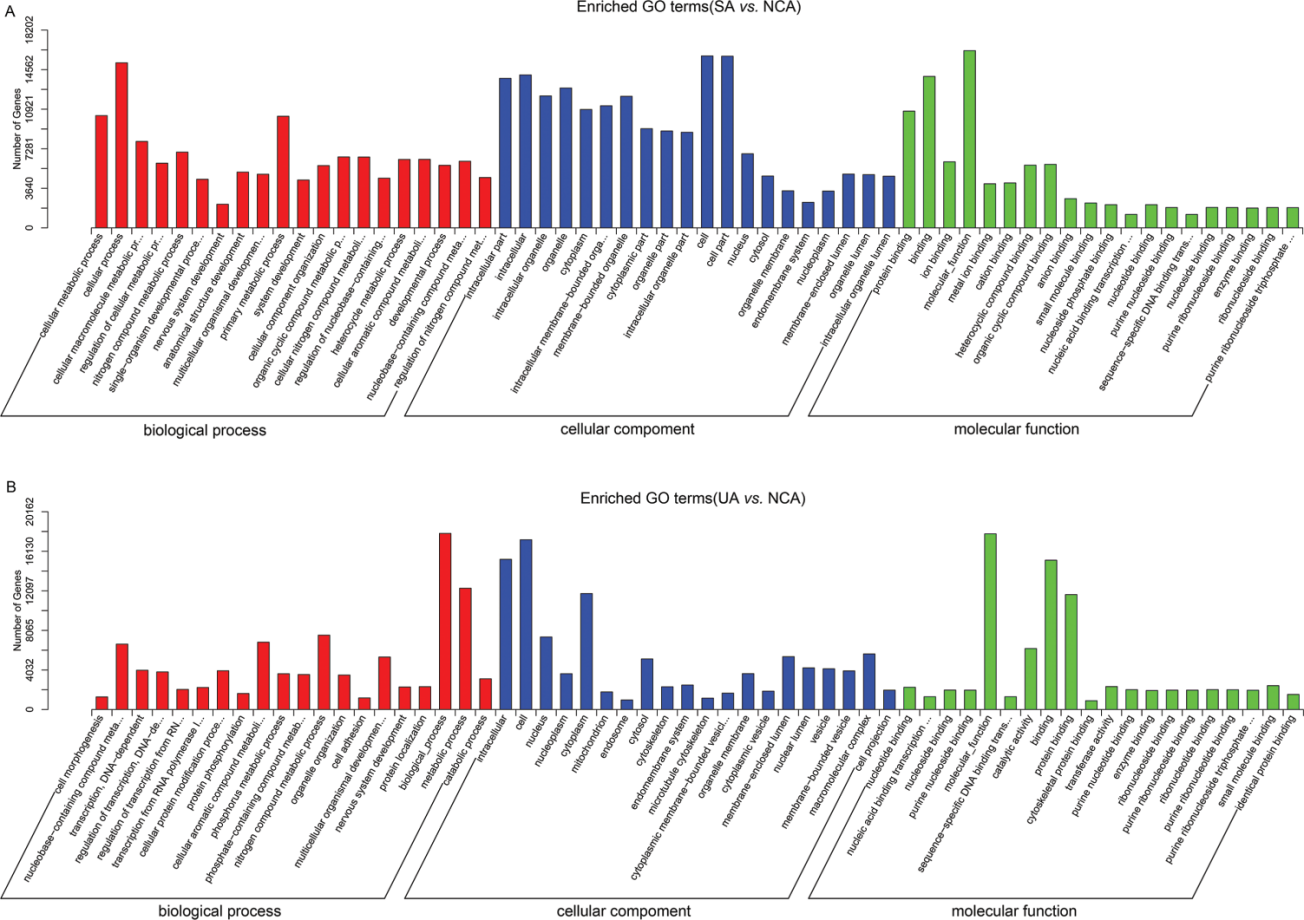
GO terms analysis of differentially expressed miRNAs in SA and UA patients compared with NCA patients. (A) Enriched GO terms of differentially expressed miRNAs in SA patients compared with NCA patients. (B) Enriched GO terms of differentially expressed miRNAs in UA patients compared with NCA patients. The top-20 GO terms of biological processes, cellular components, and molecular functions are shown. Each GO term has a corrected *p*-value <0.05. GO, gene ontology; SA, stable angina (SA); UA, unstable angina; NCA, normal coronary artery.

GO functional analysis was performed to reveal the biological functions of target genes of dysregulated miRNAs between UA, SA, and NCA patients. Target genes of dysregulated miRNAs in SA patients were involved in biological processes such as cellular metabolism, cellular macromolecule metabolism, multicellular organismal development, and other biological processes (Figure 2A), whereas in UA patients, targets of dysregulated miRNAs were associated with biological processes such as protein phosphorylation, transcription, DNA-dependent, cell adhesion (Figure 2B).

**Figure 3:**
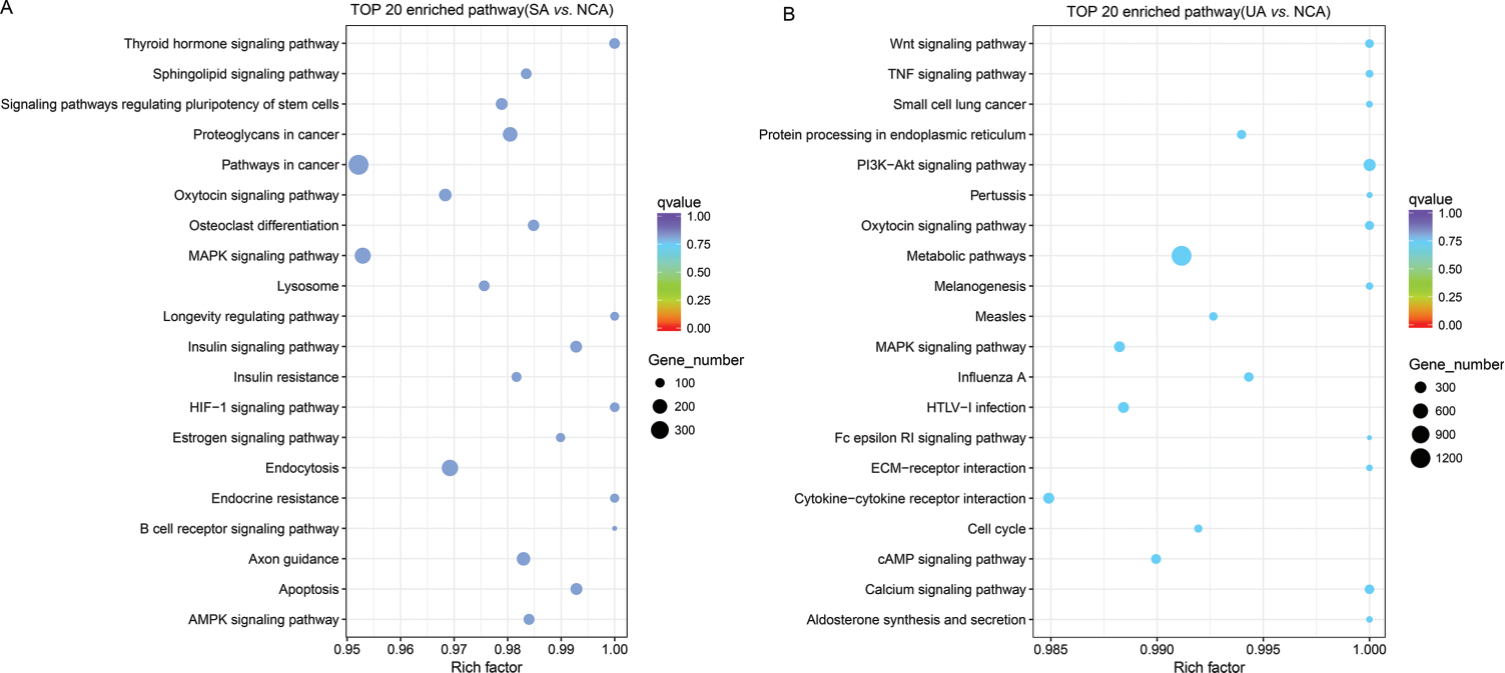
KEGG pathway analysis of differentially expressed miRNAs in SA and UA patients compared with NCA patients. (A) Enriched top-20 pathways of differentially expressed miRNAs in SA patients compared with NCA patients. (B) Enriched top-20 pathways of differentially expressed miRNAs in UA patients compared with NCA patients. Rich factor: the ratio of candidate genes enriched in the pathway to total genes in the pathway. KEGG, Kyoto Encyclopedia of Genes and Genomes; SA, stable angina (SA); UA, unstable angina; NCA, normal coronary artery.

Furthermore, KEGG analysis revealed that dysregulated miRNAs in the SA group were associated with pathways such as MAPK signaling, B cell receptor signaling, and apoptosis (Figure 3A), whereas differentially expressed miRNAs in UA group were associated with the Wnt, PI3K−Akt, and MAPK pathways (Figure 3B).

### Validation of differentially expressed miRNAs

**Figure 4:**
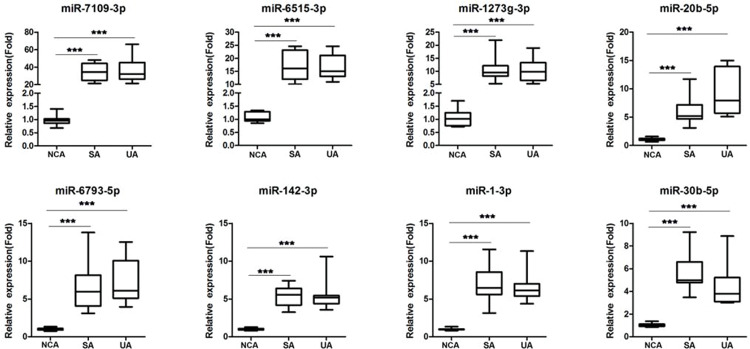
Validation of differentially expressed miRNAs by RT-qPCR. Expression patterns of eight differentially expressed miRNAs were examined in independent sets of SA (n=15), UA (n=15), and NCA (n=15) patients by RT-qPCR; statistical significance is indicated as follows: *p<0.05; **p<0.01; ***p<0.001.

To assess expression of miRNAs, we randomly selected eight miRNAs and examined their expression by RT-qPCR (Table 2). Expression of these miRNAs was assessed in independent sets of patients with UA (n=15), SA (n=15), and NCA (n=15), and the expression pattern was different in UA and SA patients as compared to that in NCA patients (Figure 4), which was consistent with the RNA sequencing results.

## DISCUSSION

We examined expression profiles of circulating miRNA in patients with UA, SA, and NCA. Our results revealed that SA and UA patients had different miRNA profiles compared with NCA patients; most dysregulated miRNAs occurred at higher levels in SA and UA patients than in NCA patients, and UA patients showed more dysregulated miRNAs than SA patients.

ACS is still one of the most severe threats to global public health (25,26). UA is a major form of ACS, however, only few specific biomarkers are available for diagnosis. Therefore, non-invasive sensitive biomarkers of UA are urgently required. miRNAs occur in body fluids and remain stable even under severe conditions (27-29). These characteristics make them ideal candidates as noninvasive biomarkers for many diseases. Recent studies suggest that miRNAs are potential diagnostic biomarkers of cardiovascular diseases such as heart failure (30) and AMI (16). A different study showed that miR-499-5p can be used to distinguish congestive heart failure from non-ST-segment elevation myocardial infarction patients (31). Moreover, miR-135a, miR-378, and miR-147 are effective biomarkers of CAD (32), and miR-208b, miR-499, and miR-1 are associated with the development of ACS and can thus be used as biomarkers of AMI (33). In the present study, we found 36 miRNAs that were dysregulated in SA patients and 161 in UA patients. These miRNAs may be attributed to the progression of CAD. Moreover, 11 miRNAs were dysregulated in both SA and UA patients. These consistently dysregulated miRNAs may be strongly associated with the development of CAD and thus appear to be particularly promising candidates for early detection of ACS.

miRNAs regulate gene expression. We, therefore, predicted the functions of differentially expressed miRNAs using GO terms classification and KEGG pathway analysis. We found that differentially expressed miRNAs regulated inflammation-associated processes such as cell adhesion and the MAPK signaling pathway (34,35). Furthermore, the Wnt pathway, which was also targeted in our study, is involved in endothelial injury, macrophage activation, and vascular smooth muscle migration, and these processes are closely associated with onset and progression of atherosclerosis (36). Previous studies showed that miR-1 is associated with cardiac injury and cardioprotection, and is increased in patients suffering from AMI (37). miR-142 is increased in atherosclerotic plaques and regulates oxLDL-induced apoptosis in macrophages; moreover, it can be used to predict major adverse cardiovascular events in CAD patients (38). In line with these observations, we observed increased levels of miR-1-3p and miR-142-3p in SA and UA patients, compared to those in NCA patients, which suggested their correlation with CAD progression.

Currently, there is no consensus regarding internal references for circulating miRNAs. U6 was used as a reference in some studies, but it easily degrades in serum (39). However, miR-16 and miR-1228 are consistently expressed in serum (40,41). In the present study, we observed that miR-16 expression varied between patients, and miR-1228 was decreased in UA patients. Recently, non-human spike-in miRNAs such as cel-miR-39 and cel-miR-67 have been commonly used as alternative references for normalization (21,23). Therefore, we used cel-miR-39 to normalize miRNA levels between different subjects.

There are some limitations of the present study which should be noted. Firstly, we profiled miRNA expression in SA and UA patients, however, we were unable to identify miRNAs that can be used to distinguish UA from SA. Secondly, white blood cells have not been examined in the present study, which may bias the generated profiles of circulating miRNAs.

In conclusion, we identified distinct circulating miRNA expression profiles in patients with UA and SA by small RNA sequencing. Dysregulated miRNAs were functionally associated with pathogenesis of CAD and are thus promising non-invasive biomarkers for early diagnosis of UA.

## AUTHOR CONTRIBUTIONS

Liu S and Guo X contributed to the data collection and manuscript writing. Weng R and Liu J contributed to data analyses. Zhong W, Weng R, Liu J and Gu X contributed to data collection. Liu S and Zhong Z conceived and planned the study, contributed to the data collection and analysis, and manuscript writing.

## Figures and Tables

**Table 1:** Clinical characteristics of patients. Means±standard deviations are shown in each case.

Variables	NCA (n=5)	SA (n=6)	UA (n=8)	*p*-value#
Age (years)	57±8.71	63±9.88	61±7.97	0.53
Sex M/F	2 (40%)	4 (66.7%)	5 (62.5%)	0.63
SBP (mmHg)	133±9.51	134.7±12.71	132.9±11.42	0.95
DBP (mmHg)	84.6±2.07	81.8±13.24	78.4±5.7	0.45
Lipid profile				
TG (mmol/L)	0.99±0.43	1.1±0.60	2.5±0.81	<0.01
TC (mmol/L)	4.43±0.91	4.41±0.74	4.43±0.63	1.00
HDL-C (mmol/L)	1.40±0.41	1.27±0.16	1.02±0.22	0.06
LDL-C (mmol/L)	2.26±0.51	2.55±0.73	2.48±0.53	0.71
Risk factors				
Active smoker (n, %)	1 (20%)	1 (16.7%)	4 (50%)	0.34
Drink (n, %)	1 (20%)	1 (16.7%)	1 (12.5%)	0.93
Hypertension (n, %)	1 (20%)	1 (16.7%)	2 (25%)	0.93
Dyslipidemia (n, %)	1 (20%)	0 (0%)	5 (62.5%)	0.04
Diabetes mellitus (n, %)	1 (20%)	0 (0%)	2 (25%)	0.43
Drug administration				
Aspirin (n, %)	2 (40%)	5 (83%)	4 (50%)	0.29
Clopidogrel (n, %)	1 (20%)	3 (50%)	5 (62.5%)	0.32
Statin (n, %)	2 (40%)	6 (100%)	7 (87.5%)	0.04
CCB (n, %)	2 (40%)	4 (67.7%)	6 (75%)	0.63
ACEI (n, %)	0 (0%)	2 (33.3%)	0 (0%)	0.09

# Comparisons between groups were performed using a one-way ANOVA for continuous variables and with a Chi-square test for categorical variables.

SA, stable angina; UA, unstable angina; NCA, normal coronary arteries; M/F, male/female; SBP, systolic blood pressure; DBP, diastolic blood pressure; TG, triglyceride; TC, total cholesterol; HDL-C, high density lipoprotein cholesterol; LDL-C, low density lipoprotein cholesterol; CCB, calcium channel blocker; ACEI, angiotensin-converting enzyme inhibitor.

**Table 2:** Dysregulated miRNAs in patients with UA and SA compared to those of NCA patients.

miRNAs	UA *vs.* NCA			SA *vs.* NCA		
	log_2_FC	*p*	Regulation	log_2_FC	*p*	Regulation
miR-7109-3p	6.28	0.0005	up	4.96	0.0020	up
miR-6515-3p	4.00	0.0025	up	4.10	0.0069	up
miR-1273g-3p	3.36	0.0013	up	3.45	0.0020	up
miR-20b-5p	3.30	7.33E-06	up	2.55	0.013	up
miR-6793-5p	2.86	0.0032	up	2.77	0.0173	up
miR-142-3p	2.45	5.19E-06	up	2.46	0.0109	up
miR-1-3p	2.44	0.0002	up	2.44	0.01	up
miR-30b-5p	1.84	0.0365	up	2.20	0.0297	up

SA, stable angina; UA, unstable angina; NCA, normal coronary artery; FC, fold change.
